# Effect of bardoxolone methyl on the lower reproductive tract microbiome in turkey breeder hens

**DOI:** 10.3389/fphys.2025.1703742

**Published:** 2025-11-17

**Authors:** Juthamas Nabthonglang, Andres Gomez, Stephanie Rutschke, Pitchaya Santativongchai, Sunantha Kosonsiriluk, Marissa Studniski, Ben Wileman, Chainarong Navanukraw, Kahina Boukherroub

**Affiliations:** 1 Department of Animal Science, University of Minnesota, St. Paul, MN, United States; 2 Department of Animal Science, Faculty of Agriculture, Khon Kaen University, Khon Kaen, Thailand; 3 Select Genetics, Willmar, MN, United States

**Keywords:** microbiome, uterovaginal junction, vagina, bardoxolone methyl, turkey

## Abstract

**Introduction:**

Fertility decline in aging turkey breeder hens is associated with reduced sperm storage in the uterovaginal junction (UVJ), inflammation, oxidative stress, and tissue aging. The mucosal microbiome is an important contributor to subfertility, with shifts in immune function, inflammation, and oxidative stress linked to microbial changes. Bardoxolone methyl, a potent activator of the nuclear erythroid 2-related factor 2 (NRF2) pathway, enhances antioxidant defenses and reduces inflammation. This study investigated if bardoxolone methyl treatment alters the microbial composition and diversity of the UVJ and vagina in turkey hens.

**Methods:**

Forty turkey hens (59 weeks old) were randomly assigned to a bardoxolone methyl group (n = 20) or a control group (n = 20). Birds received intramuscular tail injections of bardoxolone methyl or vehicle, every other day for two weeks. Swabs from the UVJ and vagina (VAG) were collected for 16S rRNA sequencing. Microbial diversity, differential taxonomic composition, and predicted functional pathways were assessed using QIIME2, PICRUSt2, and R-based statistical packages. Microbiome profiles revealed significant differences between UVJ and VAG communities.

**Results:**

The VAG showed higher bacterial richness, while both sites were dominated by *Firmicutes, Proteobacteria, Thermoproteota, and Actinobacteriota phyla*, indicator species analyses identified enrichment of *Staphylococcus* and *Escherichia* in UVJ, and Lactobacillaceae in VAG. Bardoxolone methyl did not significantly alter global alpha diversity but selectively modulated unweighted beta diversity and low-abundance taxa, enriching *Corynebacterium* in UVJ and rare taxa like *Armatimonadota* and *Omnitrophota* in the VAG. Functional predictions indicated bardoxolone methyl’s association with enrichment of pathways including energy metabolism, nucleotide biosynthesis, protein quality control, and redox balance, particularly in the UVJ.

**Discussion:**

This study provides the first characterization of the turkey lower reproductive tract microbiome, revealing tissue-specific communities and functional profiles between the UVJ and vagina. Bardoxolone methyl treatment did not alter overall microbial diversity, but selectively enriched low-abundance taxa and metabolic pathways related to energy metabolism, nucleotide biosynthesis, and stress resilience, particularly in the UVJ. These findings indicate that bardoxolone methyl treatment can finetune microbial functional capacity without destabilizing overall community structure. The results also highlight the importance of considering tissue-specific differences and functional potential when investigating reproductive function.

## 1 Introduction

The United States has the largest turkey industry globally, with turkey meat ranking as the second most consumed poultry meat within the country (Food and Agriculture Organization of the United Nations, 2023). Commercial turkey production depends on breeder hens to produce fertilized eggs, which are subsequently hatched and reared for meat production. However, fertility in turkey breeder hens naturally declines at the later stages of the laying cycle, typically leading to reduced hatchability and productivity in later stages of the laying cycle. Therefore, improving or sustaining reproductive performance in aging breeder hens is critical to optimize flock productivity and maintain economic viability.

The primary factor contributing to fertility decline in aging turkey breeders is their reduced ability to retain sperm within sperm storage tubules (SSTs), specialized tubular structures located at the uterovaginal junction (UVJ) of the reproductive tract (Christensen, 1981; Sasanami et al., 2013). Research indicates that this decline is associated with dysregulation in the sperm selection and storage processes within the vagina and UVJ, respectively. Changes in the immune cell composition and localization, increased inflammation, and accelerated tissue aging have been observed towards the end of laying cycle, when fertility is typically at its lowest (Kosonsiriluk et al., 2023; Chen et al., 2024). These processes are known to increase the production of reactive oxygen species (ROS), leading to oxidative stress within tissues, while simultaneously reducing the intrinsic antioxidant capacity of the cells (Khansari et al., 2009; Biswas, 2016). Encouragingly, reducing oxidative stress and increasing the antioxidant capacity of cells within reproductive tissues is achievable through the use of readily available, naturally derived compounds that activate the Nuclear Factor Erythroid 2-related Factor 2 (NRF2) pathway, such as bardoxolone methyl (Ruiz et al., 2013; Pei et al., 2019; Robledinos-Antón et al., 2019).

Changes in the immune system, inflammation, and oxidative stress have also been associated with changes in the microbiome (Kim et al., 2006; Witkin et al., 2007; Marciano and Vajro, 2017). Recent studies have shown that gut epithelial cells rapidly produce ROS in response to enteric commensal bacteria (M Jones et al., 2012), and similar ROS responses occur in other cell types exposed to microbial signals (O'Connell et al., 2005). These physiologically generated ROS act as second messengers in signaling pathways triggered by proinflammatory cytokines and growth factors, thereby modulating cellular activity (Witkin et al., 2007; Marciano and Vajro, 2017).

The reproductive tract microbiome has also been implicated in subfertility across multiple species including humans, mice, cows, swine, and ewes (García-Velasco et al., 2017; Deng et al., 2019; Chen et al., 2020; Sanglard et al., 2020). By contrast, no published studies have examined the relationship between microbiome and fertility in avian species. Among the limited research on avian reproductive tract microbiomes, only one study has specifically investigated the vaginal microbiome in chickens, and none have characterized the microbial composition of the turkey UVJ or vagina (Lee et al., 2019; Shterzer et al., 2020; Wen et al., 2021; Kursa et al., 2022). Furthermore, to our knowledge, no studies have attempted to alter the microbial composition of the lower reproductive tract in avian species.

The objective of the present study was to determine if administration of a NRF2-activating compound to turkey hens alters the microbial composition and diversity of the UVJ and vagina. This study provides the first characterization of the vaginal and UVJ microbiome in turkeys, both with and without antioxidant treatment, as well as the associated changes in microbial composition and pathway responses to the treatment.

## 2 Materials and methods

### 2.1 Experimental design

**FIGURE 1:**
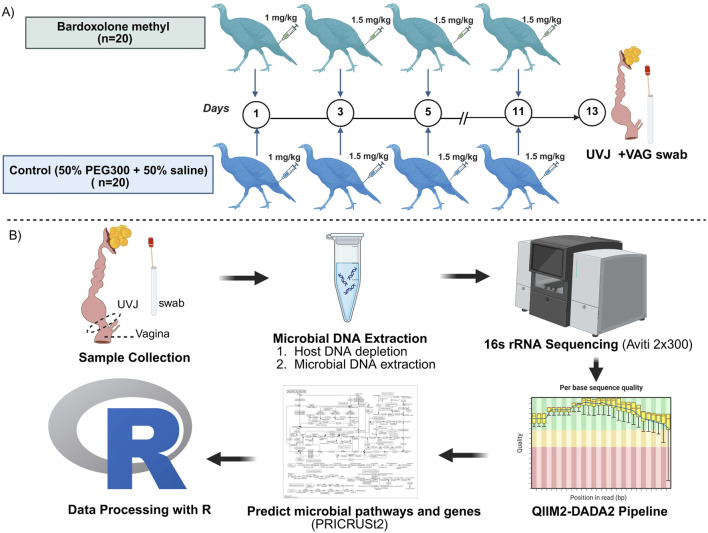
**(A)** Experimental designs and **(B)** Workflow for sample collection, DNA extraction, sequencing, and data analysis. Turkey hens (n = 40) were randomly assigned to bardoxolone methyl (BAR) and control (CON) groups. Uterovaginal junction (UVJ) and vaginal (VAG) swabs were collected on day 14 post-injection with bardoxolone methyl or diluent (PEG300 + saline), then snap-frozen for microbial DNA extraction. DNA concentrations averaged 2.3 ± 1.4 ng/μL. The V4 region of the 16S rRNA gene was amplified by polymerase chain reaction (PCR) and sequenced. Sequences were processed using QIIME2 pipeline with DADA2 plugin for denoising and amplicon sequence variant (ASV) identification. Taxonomic assignments were made using the Greengenes 2023.2 database. Data filtering and downstream statistical analyses, including diversity analysis, indicator species analysis, and prediction of pathways and genes were performed in R using Wilcoxon, PERMANOVA, and ANOSIM. Created in BioRender. Boukherroub K. (2025) https://BioRender.com/a47vgpq.

A total of 40 turkey breeder hens (*Meleagris gallopavo*), aged 59 weeks (28th week of lay) at the end of the production cycle were kindly donated by a Minnesota turkey breeding company. The hens were housed under standard commercial conditions with *ad libitum* access to a conventional diet containing 18% protein and water. All animal care procedures were approved by the Institutional Animal Care and Use Committee (IACUC) at the University of Minnesota under protocol number 2401-41696A. Hens were randomly assigned to two groups: bardoxolone methyl (BAR; n = 20) and control (CON; n = 20). The BAR group received an initial intramuscular injection of 1 mg/kg bardoxolone methyl (HY-13324, purity ≥98%, MedChemExpress LLC., NJ, United States), dissolved in the diluent (50% polyethylene glycol 300 (PEG300) and 50% saline), administered in the tail area. Subsequent injections of 1.5 mg/kg bardoxolone methyl in the diluent were given every other day for 2 weeks. The CON group received equal volumes of the diluent without bardoxolone methyl, following the same injection schedule (Figure 1A). The bardoxolone methyl dose was derived from allometric scaling of the human therapeutic range (0.3–2 mg/kg/day (Nangaku et al., 2020). Based on the average body weight of turkey hens (30–35 lb; ∼14.5 kg), this corresponded to an equivalent exposure of 0.9–5.96 mg/kg per injection. The initial low dose was used to evaluate potential local immune or inflammatory reactions; the site was monitored closely, and dosing adjustments were made in consultation with the attending veterinarian. Because turkeys are particularly sensitive to pharmacologic interventions, the final regimen was maintained within the low-to-moderate range of the scaled therapeutic window to balance safety and efficacy, promoting sustained NRF2 activation while minimizing adverse effects.

### 2.2 Sample collection

Following a 2-week treatment period, hens were humanely euthanized using assisted mechanical cervical dislocation in accordance with approved animal care protocols. The lower reproductive tract was excised by dissection from the uterus to the cloacal opening. Vaginal tissue was sampled from the mid-region of the vagina 1 cm distal to the UVJ. Subsequently, the mucosal surfaces of the excised 1 x 1.5 cm UVJ and vagina (VAG) sections were swabbed using sterile swabs. Swabs were immediately transferred into sterile microcentrifuge tubes and stored at −80 °C until DNA extraction and microbial analysis (Figure 1B).

### 2.3 DNA extraction and 16s rRNA gene sequencing

Microbial DNA was extracted from the UVJ and VAG tissue swabs using the HostZERO™ Microbial DNA Kit (Zymo Research, California, United States), following the manufacturer’s instructions to reduce host DNA contamination. The extracted DNA concentrations were 2.4 ± 1.4 ng/μL across samples as measured using a NanoDrop One Microvolume UV-Vis Spectrophotometer (Thermo Fisher Scientific, Waltham, MA). The V4 region of the 16S rRNA gene was amplified using primers 515F (GTGYCAGCMGCCGCGGTAA) and 806R (GGACTACNVGGGTWTCTAAT) using the dual index approach (Gohl et al., 2016), and libraries were paired-end sequenced (2 × 300 bp) on the AVITI platform at the University of Minnesota Genomics Center (UMGC).

Raw reads (obtained after sequencing) were subjected to quality control using FASTQC (Andrews et al., 2010) and Cutadapt, which was used to trim adapter, primer sequences, and low quality bases (Martin, 2011). Sequences, that were too short were filtered out. High quality sequences were processed using QIIME2 version 2023.2 (Bolyen et al., 2019) and amplicon sequence variants (ASVs) were determined with the DADA2 plug in using the denoise-paired method (Shterzer et al., 2024; Callahan et al., 2016). Greengenes2 (version 2022.10) was used as the reference database for the taxonomic assignment of ASVs. Potential contaminants were removed using the Decontam package (Callahan and Davis, 2025). ASVs with fewer than 5 total reads across the entire dataset and those present in fewer than 3 samples were filtered out using the labdsv package (Roberts, 2019). Functional predictions of 16S rRNA data was performed via PICRUSt 2 (Langille et al., 2013).

### 2.4 Statistical analysis

Further analyses including alpha diversity metrics (observed ASVs, Shannon, and Simpson diversity index), beta diversity (Bray-Curtis dissimilarity), and indicator species analyses were carried out using the R program and the phyloseq (McMurdie and Holmes, 2013), labdsv (Roberts, 2019), ape (Paradis et al., 2019) and vegan (Oksanen et al., 2025) packages.

All graphs were generated using the ggplot2 (Wickham et al., 2025) and pheatmap (Kolde, 2025) packages in R. Bar plots were constructed showing the relative abundance of taxa at the phylum, family, and genus levels. Violin plots were generated to visualize the distribution of indicator species, pathways, and gene abundances across treatment or tissue sites. Heatmaps of the top abundant pathways and genes were also constructed to highlight patterns of functional variation across samples. Pathway and gene prediction of microbial metabolic pathways was performed using PICRUSt2 (Douglas et al., 2020), and pathway-level differences were further analyzed using indicator species analysis with pathway annotation based on MetaCyc and KEGG databases.

Statistical analyses for alpha and beta diversity comparisons, as well as relative abundance comparisons, were primarily performed using the Wilcoxon rank-sum test to identify statistically significant differences in ASV, pathway, and gene abundances across the following comparisons: UVJ vs. VAG regardless of treatment; BAR vs. CON regardless of tissue type; BAR vs. CON within UVJ; and BAR vs. CON within VAG. Beta diversity analysis was conducted using Bray-Curtis dissimilarity, and group differences were assessed using both permutational multivariate analysis of variance (PERMANOVA) and analysis of similarities (ANOSIM). For variables with normally distributed data as determined by the Shapiro-Wilk test, additional parametric tests such as t-test and analysis of variance (ANOVA) were applied. A *P*-value <0.05 was considered statistically significant. Differential abundance analyses were performed using Wilcoxon rank-sum tests. Differences were considered statistically significant at *P* < 0.05.

## 3 Results

### 3.1 Distinct microbial diversity and taxonomic composition between the UVJ and vagina

**FIGURE 2:**
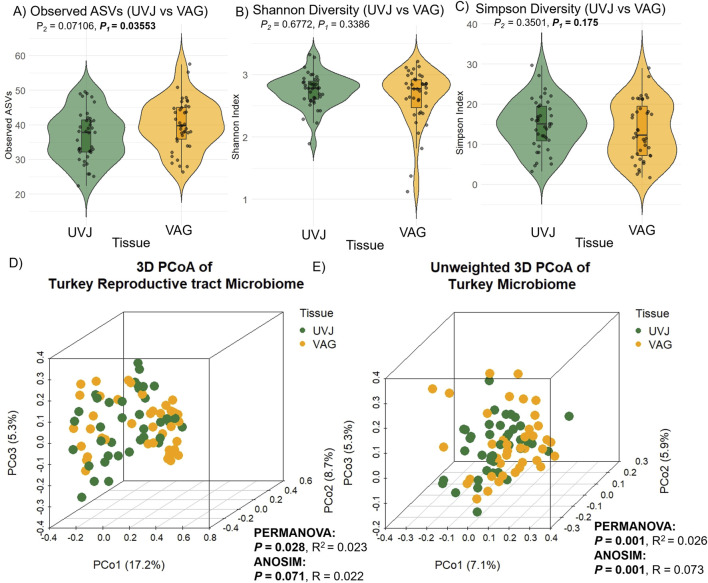
Alpha and beta diversity of turkey microbiota from the uterovaginal junction (UVJ) and vaginal (VAG) tissues. Alpha diversity indices between tissue types: **(A)** Observed ASVs, **(B)** Shannon diversity indices, and **(C)** Simpson diversity indices. A significant difference was observed in observed ASVs (one-tailed *P* = 0.0359), while Shannon and Simpson indices showed no significant differences. Beta diversity visualized by principal coordinate analysis (PCoA) based on **(D)** Bray-Curtis distance and **(E)** unweighted Bray-Curtis distance. Tissue type significantly influenced microbial composition (PERMANOVA: weighted *P* = 0.028; unweighted *P* = 0.001). ANOSIM results showed consistent trends. *P*_1_ = one-tailed p-values; *P*_2_ = two-tailed p-values.

Microbial composition analysis revealed significantly higher bacterial richness in the VAG compared to the UVJ irrespective of treatment group as indicated by the number of observed ASVs (Wilcoxon one-tailed test, *P* = 0.0359; Figure 2A). In contrast, the Shannon (which accounts for both richness and evenness) and Simpson diversity indices (which reflects species dominance) showed no significant differences, suggesting comparable community evenness (Figures 2B,C). ANOVA results supported this pattern, confirming a tissue-specific effect on richness (*P* = 0.045) without affecting overall diversity indices (Table 1).

**TABLE 1:** Analysis of variance (ANOVA) for alpha diversity indices (Observed species, Shannon, and Simpson) and beta diversity (Weighted and Unweighted Bray-Curtis) in turkey samples.

Diversity index	Source	*P*-value
Alpha diversity		
- Observed ASVs	Treatment	0.867
	Tissue	0.045^a^
	Treatment × Tissue	0.963
- Shannon	Treatment	0.327
	Tissue	0.615
	Treatment × Tissue	0.827
- Simpson	Treatment	0.137
	Tissue	0.330
	Treatment × Tissue	0.974
Beta diversity		
- Weighted Bray-Curtis	Treatment	0.350
	Tissue	0.025^a^
	Treatment × Tissue	0.984
- Unweighted Bray-Curtis	Treatment	0.140
	Tissue	0.001 ***
	Treatment × Tissue	0.215

^a^ Significant at *P* < 0.05.

Multivariate analysis based on Bray-Curtis dissimilarity revealed distinct microbial communities between UVJ and VAG, regardless of treatment. PCoA showed significant clustering or separation between tissue types (Figures 2D,E), which was confirmed via PERMANOVA for both weighted (*P* = 0.028; R^2^ = 0.023) and unweighted (*P* = 0.001; R^2^ = 0.026) distances (Table 1). The lower P-value and slightly greater R^2^ between tissue types in the unweighted analysis may suggest that differences in microbiome composition between UVJ and VAG are driven by low-abundance taxa. ANOSIM further supported compositional dissimilarity between UVJ and VAG microbiomes, with both weighted (*P* = 0.071, R = 0.022) and unweighted ANOSIM (*P* = 0.001, R = 0.073) indicating minor but significant differences, particularly for the unweighted analysis.

**FIGURE 3:**
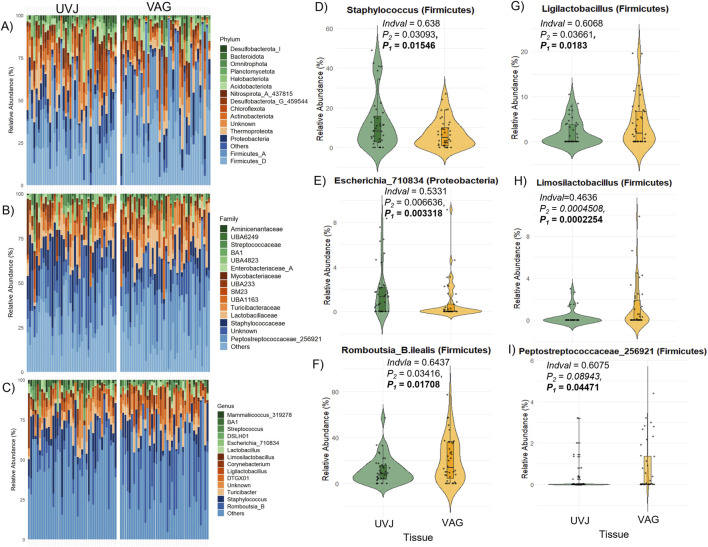
Taxonomic composition and differentially abundant genera between the UVJ and vagina in turkeys. **(A–C)** Relative abundance of microbial taxa at the phylum levels, grouped by tissue type (UVJ vs. VAG). **(D–I)** Violin plots showing the relative abundance of selected genera or taxa that significantly differed between tissues, including: *Staphylococcus* (Firmicutes), *Escherichia_710834* (Proteobacteria), *Romboutsia_B.ilealis* (Firmicutes), *Ligilactobacillus* (Firmicutes), *Limosilactobacillus* (Firmicutes), and *Peptostreptococcaceae_256921* (Firmicutes).

Taxonomic profiling highlighted tissue-specific differences at multiple taxonomic levels. Both sites were dominated by the phyla *Firmicutes, Proteobacteria, Thermoproteota*, and *Actinobacteriota* (Figures 3A–C), with *Peptostreptococcaceae_256921*, *Staphylococcaceae*, *Lactobacillaceae*, and *Enterobacteriaceae_A* among the most abundant families, and *Mammaliicoccus_319278*, *Streptococcus*, *Lactobacillus*, *Ligilactobacillus*, *Limosilactobacillus*, *Escherichia_710834*, and *Romboutsia_B* as the predominant genera (Supplementary Figures S1A,B). At the ASV level, indicator species analyses revealed that *Staphylococcus and Escherichia_710834* were enriched in UVJ samples, while *Romboutsia_B.ilealis, Ligilactobacillus*, and *Limosilactobacillus* were more abundant in the VAG (Figures 3D–I). The relative abundances of other discriminatory taxa across tissues are summarized in Table 2, reflecting distinct microbial niches along the turkey reproductive tract.

**TABLE 2:** Relative abundance of discriminatory bacteria across tissue.

Taxa	Abundance in UVJ	Abundance in vagina	Indicator value	Wilcoxon p-value
*s__Limosilactobacillus.ingluviei (Firmicutes_D)*	0.2421 ± 0.7789	**0.9026** ± **1.6968**	0.3745	0.00188
*s__Limosilactobacillus.fermentum (Firmicutes_D)*	**0.7926** ± **1.6971**	0.2259 ± 0.8241	0.2724	0.01925
*g__Anaerococcus (Firmicutes_A)*	0.000 ± 0.000	**0.4785** ± **1.3771**	0.2500	0.00083
*g__Clostridium (Firmicutes_A)*	0.0134 ± 0.0849	**0.1174** ± **0.4067**	0.2243	0.00433
*g__Enterococcus (Firmicutes_D)*	0.0616 ± 0.3894	**0.535** ± **2.1398**	0.2242	0.01236
*s__UTCFX2.sp002050125 (Chloroflexota)*	**0.3207** ± **0.992**	0.000 ± 0.000	0.1500	0.04418
*f__UBA6902 (Nitrospirota)*	**0.3321** ± **1.5605**	0.000 ± 0.000	0.1500	0.01181
*f__UBA6249 (Omnitrophota)*	0.000 ± 1e-04	**0.2726** ± **0.8603**	0.1500	0.04629
*f__Ruminococcaceae (Firmicutes_A)*	0.000 ± 0.000	**0.0518** ± **0.2025**	0.1250	0.02249

Abundance data are reported as the mean ± standard deviation. Numbers in bold show which tissue has higher abundance.

Taken together, these results indicate that the UVJ and vagina harbor distinct microbial communities not only in terms of diversity but also taxonomic composition, with specific bacterial genera preferentially colonizing each anatomical site.

Additionally, microbiota diversity was compared between BAR and CON groups irrespective of site (UVJ or VAG). No significant differences were observed in alpha diversity (observed ASVs, Shannon index), though Simpson diversity showed a non-significant trend toward lower diversity in the CON group (*P* = 0.0797; Supplementary Figures S2A–C). Beta diversity analysis did not show significant group clustering or separation (*P* > 0.05; Supplementary Figures S2D,E), suggesting BAR did not broadly alter microbial diversity. However, ASV-level indicator species analysis revealed minor differential enrichment of specific taxa between BAR and CON regardless of tissue. For example, *Bin103. sp002238925 (Spirochaetota)* and *Varibaculum (Actinobacteriota)* were higher in BAR, while *Anaerolineales (Chloroflexota)* and *Ligilactobacillus (Firmicutes)* were enriched in CON (Wilcoxon test, *P* < 0.05; Supplementary Figures S3), indicating selective modulation by BAR despite stable overall diversity.

### 3.2 Predicted microbial pathways and genes differ between UVJ and vaginal microbiomes

**FIGURE 4:**
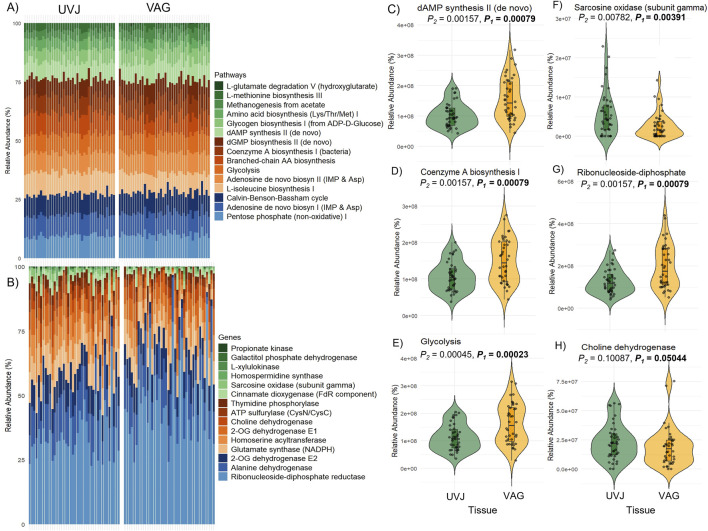
Predicted microbial metabolic pathways and genes in the uterovaginal junction (UVJ) and vaginal (VAG) tissues. **(A)** Stacked bar plot showing the relative abundances of the top 15 predicted MetaCyc metabolic pathways in UVJ and VAG samples based on PICRUSt2 analysis. **(B)** Stacked bar plot showing the top 15 predicted genes contributing to microbial metabolic pathways in both tissues. **(C–E)** Violin plots showing the relative abundances of selected pathways that differed significantly between UVJ and VAG tissues **(F–H)** Violin plots of gene families associated with significantly different pathways.

Predicted microbial metabolic pathways and specific genes as revealed via PICRUSt2 indicated distinct functional profiles between the UVJ and VAG microbiomes. Overall, the most abundant predicted pathways in UVJ and vagina included the Pentose phosphate (non-oxidative) I, Adenosine *de novo* biosynthesis I (IMP & Asp), and the Calvin-Benson-Bassham cycle (Figure 4A), reflecting core microbial metabolic functions. At the gene level, highly represented functions included ribonucleoside-diphosphate reductase, Alanine dehydrogenase, 2-OG dehydrogenase E2, glutamate synthase, and ATP sulfurylase, among others (Figure 4B), further emphasizing conserved microbial processes across tissues. However, indicator species analysis identified several pathways that were significantly enriched in specific tissue types. Notably, dAMP synthesis II (*de novo*), Coenzyme A biosynthesis, and Glycolysis pathways were significantly more abundant in the VAG microbiome (*P* < 0.001 for all; Figures 4C–E), suggesting enhanced nucleotide and energy metabolism in this region.

In contrast, UVJ samples showed higher levels of specific predicted genes, such as the sarcosine oxidase gene (*soxG*, subunit gamma), indicating tissue-specific microbial functions related to amino acid metabolism (Figure 4F). Additionally, genes involved in ribonucleoside-diphosphate biosynthesis were enriched in VAG samples (Figure 4G), while choline dehydrogenase gene (*chdh*) tended to be more abundant in UVJ, though this difference was marginally significant (P = 0.05; Figure 4H). Together, these findings highlight both shared core metabolic pathways and site-specific gene-level metabolic potentials along the lower reproductive tract.

### 3.3 Impact of bardoxolone methyl on microbial diversity and predicted functions in the UVJ

**FIGURE 5:**
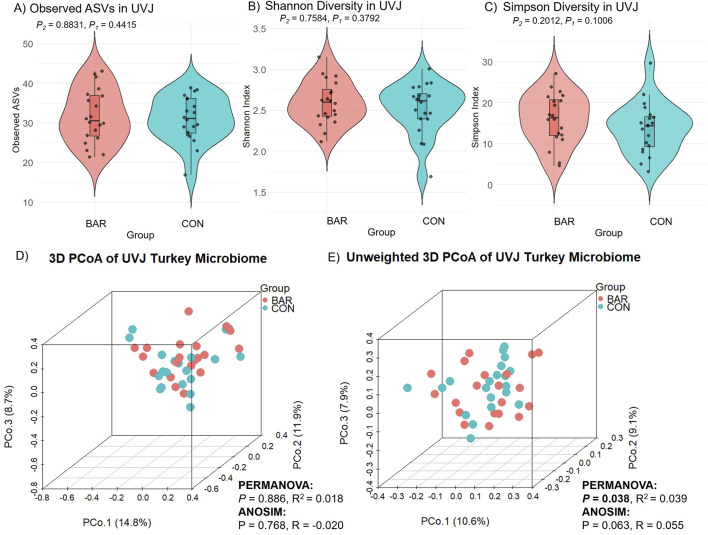
Alpha and beta diversity analyses of the uterovaginal junction (UVJ) microbiome in the bardoxolone methyl (BAR) and control (CON) groups. Alpha diversity metrics, including **(A)** observed ASVs, **(B)** Shannon diversity indices, and **(C)** Simpson diversity indices, revealed no significant differences between treatment groups. Beta diversity was visualized using principal coordinate analysis (PCoA) based on **(D)** Bray-Curtis dissimilarity and **(E)** unweighted Bray-Curtis distances.

The effect of bardoxolone methyl on the UVJ microbiome was evaluated through taxonomic and functional analyses. Alpha diversity metrics (observed ASVs, Shannon, and Simpson indices) showed no significant difference between BAR and CON groups, indicating stable species richness and evenness (Figures 5A–C). Although weighted distances showed no difference (*P* > 0.05; Figure 5D), unweighted beta diversity (Bray–Curtis) analyses revealed modest but significant compositional clustering (PERMANOVA P = 0.038, R^2^ = 0.039; ANOSIM P = 0.063, R = 0.055), suggesting BAR may influence low-abundance taxa (Figure 5E).

**FIGURE 6:**
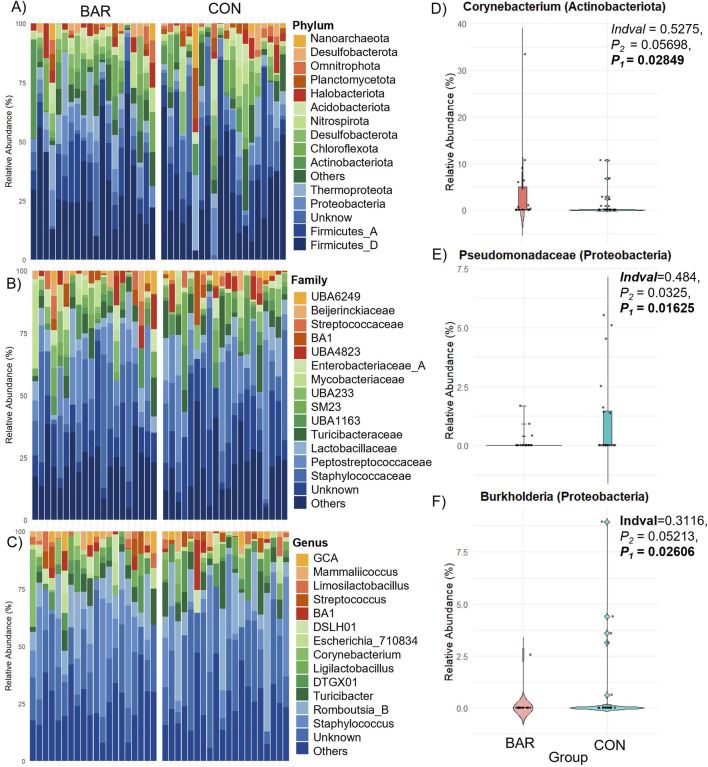
Taxonomic composition and differential abundance of the UVJ microbiome in turkeys treated with BAR or CON. **(A–C)** Stacked bar plots show the relative abundance of bacterial taxa at the phylum, family, and genus levels. **(D–F)** Differentially abundant taxa between groups were identified, with significantly higher relative abundance of *Corynebacterium* (Actinobacteriota) in the BAR group **(D)**, and *Pseudomonadaceae* **(E)** and *Burkholderia* **(F)** (both Proteobacteria) in the CON group.

Dominant phyla in the UVJ across BAR and CON groups included *Firmicutes, Proteobacteria, Thermoproteota,* and *Actinobacteriota.* Families such as *Staphylococcaceae* and *Peptostreptococcaceae* were prevalent, with key genera including *Staphylococcus, Romboutsia_B.ilealis, Turicibacter, Corynebacterium,* and *Ligilactobacillus* (Figures 6A–C). Indicator species analysis identified ASVs from *Corynebacterium* as significantly enriched in BAR, while *Pseudomonadaceae* and *Burkholderia* were more abundant in CON group (Figures 6D–F), suggesting selective promotion of potentially beneficial taxa and suppression of potentially pro-inflammatory ones by BAR.

**FIGURE 7:**
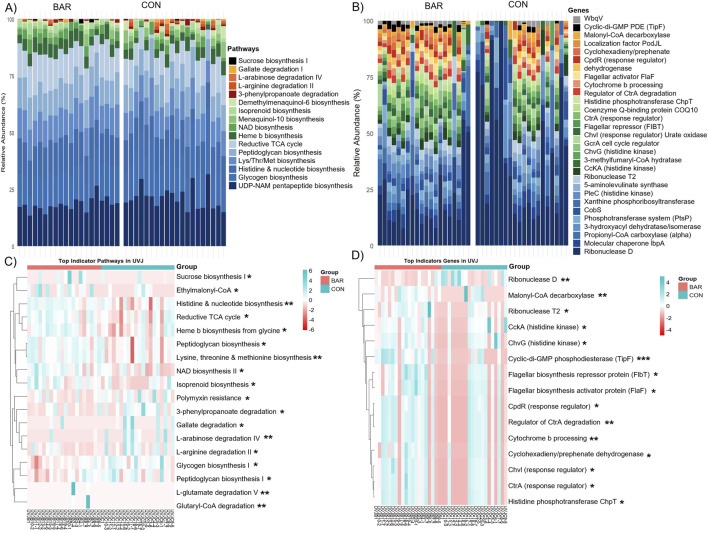
Predicted microbial metabolic pathways and genes in the uterovaginal junction (UVJ) in the bardoxolone methyl-treated (BAR) and control (CON) groups. Stacked bar plot showing the relative abundance of predicted MetaCyc **(A)** pathways and **(B)** genes. Heatmaps displaying the top indicator functional **(C)** pathways and **(D)** genes associated with BAR and CON groups identified through indicator species analysis based on PICRUSt2 prediction. Significance levels: *P* < 0.05 (*), *P* < 0.01 (**), and *P* < 0.001 (***).

Functional inference analysis was then conducted to identify metabolic consequences associated with these taxonomic changes. Microbial metabolic potential was predicted based on pathway and gene-level analyses. BAR-treated samples exhibited an increased relative abundance of microbial pathways involved in carbohydrate and nucleotide metabolism, including sucrose biosynthesis I, NAD biosynthesis, histidine biosynthesis, nucleotide biosynthesis, and the reductive tricarboxylic acid (TCA) cycle (Figures 7A,B). At the gene level, BAR samples exhibited higher abundance of regulatory genes, including Cell cycle kinase A (*CckA*), Chromosomal virulence histidine kinase G (*ChvG*), Chromosomal virulence response regulator I (*ChvI*), Cyclic-di-GMP phosphodiesterase F (*TipF*), which are associated with environmental sensing and microbial interactions (Figures 7C,D).

Despite these functional enrichment, overall pathway- and gene-level alpha and beta diversity remained unchanged between BAR and CON groups in the UVJ (Supplementary Figures S5A–E, S5A–E), indicating targeted or selected microbial modulation without widespread disruption. These findings suggest that BAR supplementation selectively alters the UVJ microbiome by promoting a potentially beneficial microbial environment and enhancing functional pathways associated with energy metabolism and environmental adaptability.

### 3.4 Impact of bardoxolone methyl on microbial diversity and predicted functions in the vagina

**FIGURE 8:**
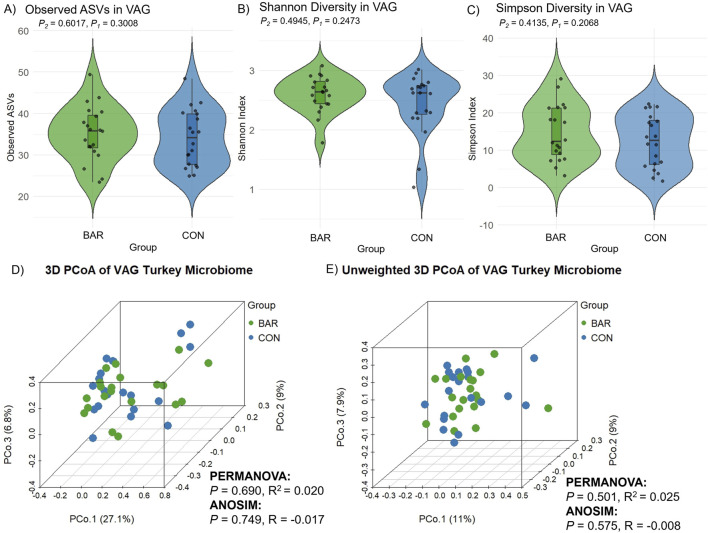
Alpha and beta diversity of the vaginal microbiome (VAG) in the bardoxolone methyl-treated (BAR) and control (CON) groups. Alpha diversity metrics including **(A)** observed ASVs, **(B)** Shannon diversity indices, and **(C)** Simpson diversity indices, showed no significant differences between groups (Wilcoxon test, *P* > 0.05). Principal coordinate analysis (PCoA) of beta diversity based on **(D)** Bray–Curtis dissimilarity and **(E)** unweighted Bray–Curtis distance. No significant clustering by treatment group was observed in either analysis (PERMANOVA and ANOSIM, *P* > 0.05).

**FIGURE 9:**
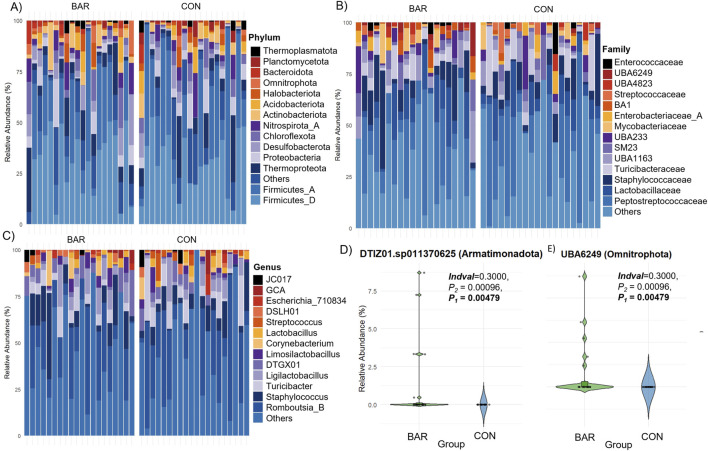
Taxonomic composition of the vaginal microbiome in the bardoxolone methyl-treated (BAR) and control (CON) groups. Stacked bar plots showing relative abundance at the **(A)** phylum, **(B)** family, and **(C)** genus levels across individual samples. Each bar represents one sample. **(D)** Differential abundance of specific ASVs significantly enriched in the BAR group.

In contrast to the UVJ, BAR treatment had minimal effect on vaginal microbial diversity. Alpha and beta diversity metrics showed no significant differences between groups (Figures 8A–E), indicating compositional stability. Taxonomic profiling revealed *Firmicutes, Thermoproteota,* and *Proteobacteria* as dominant phyla in the vagina across both CON and BAR groups (Figure 9A). At the family level, *Peptostreptococcaceae, Lactobacillaceae,* and *Staphylococcaceae* were predominant (Figure 9B), with *Limosilactobacillus, Ligilactobacillus,* and *Streptococcus* emerging as the most abundant genera (Figure 9C).

However, indicator species analysis identified two low-abundance ASVs significantly enriched in the BAR group: *DTIZ01. sp011370625 (Armatimonadota)* and *UBA6249 (Omnitrophota)*, both showing higher relative abundance in the vagina of BAR-treated hens compared to CON animals (Figure 9D). These results suggest that BAR selectively modulates specific low-abundance taxa without affecting overall community structure, consistent with patterns observed in the UVJ.

To assess functional implications, predicted microbial metabolic pathways and gene profiles were analyzed. Although overall diversity of KEGG Ortholog pathways and gene functions did not differ significantly between groups (Supplementary Figures S6A–E), deeper analysis revealed distinct functional enrichments in the BAR group.

**FIGURE 10:**
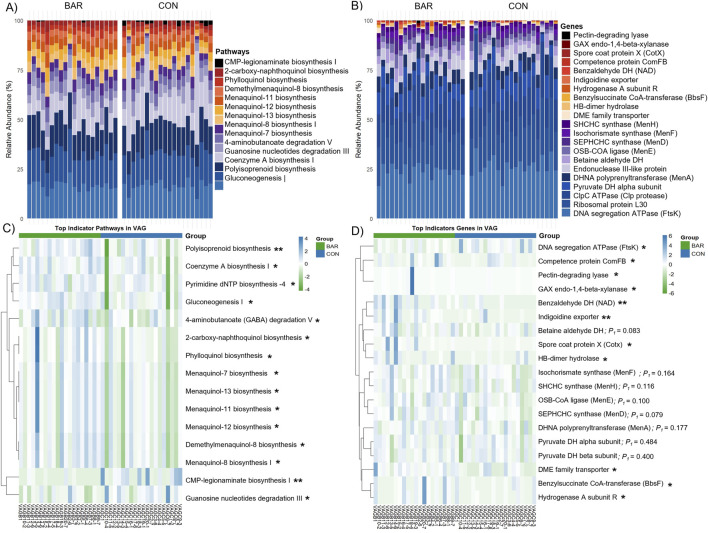
Predicted microbial metabolic pathways and genes of the vaginal microbiome of the bardoxolone methyl-treated (BAR) and control (CON) groups. Stacked bar plot showing the relative abundance of predicted microbial **(A)** pathways and **(B)** genes. Heatmaps highlight differentially abundant **(C)** pathways and **(D)** genes, with several functions enriched in the BAR group. *P < 0.05; *P < 0.01.

Specifically, several pathways were enriched, including gluconeogenesis I, polyisoprenoid biosynthesis, coenzyme A biosynthesis, and guanosine nucleotides degradation III (Figure 10A) in the BAR group. These pathways are involved in energy metabolism, nucleotide turnover, and membrane biosynthesis, suggesting enhanced metabolic activity. At the gene level, BAR-treated birds showed increased abundance of genes associated with DNA replication (e.g., *FtsK*), translation (ribosomal protein L30), central carbon metabolism (pyruvate dehydrogenase alpha subunit, *pdhA*), and protein quality control (*ClpC* ATPase) (Figure 10B). Indicator analysis further confirmed enrichment of genes such as pyruvate dehydrogenase (*pdhA* and *pdhB* subunits) and betaine aldehyde dehydrogenase (*betA*) (*P* < 0.01), potentially pointing to improved microbial energy production and stress resilience in the BAR group (Figures 10C,D). Additional enriched functions in BAR treated animals included genes related to membrane transport and redox regulation, such as the indigoidine exporter and menaquinone biosynthesis enzymes (*MenF/MenH*).

## 4 Discussion

This study provides the first comprehensive characterization of the microbial communities in the lower reproductive tract of turkey hens, focusing on the vagina and the UVJ, and further evaluates their modulation by the potent NRF2 activator bardoxolone methyl. We found that the microbiome composition and predicted metabolic functions differed markedly between the UVJ and vagina, with the vagina harboring greater bacterial richness and distinct community structures compared with the UVJ. Administration of BAR did not significantly affect global microbial alpha diversity but was associated with selective enrichment of low-abundance taxa and functional pathways, particularly in the UVJ. These findings reveal previously unexplored host–microbe interactions in the avian reproductive tract and provide new insights into how antioxidant-mediated NRF2 activation may influence microbial functional capacity without destabilizing overall community structure.

Consistent with their distinct physiological roles, the UVJ and vagina harbored significantly different microbial communities. We observed that vagina exhibited greater bacterial richness compared with UVJ, while overall Shannon and Simpson indices were similar, suggesting comparable evenness across tissues. Beta diversity analyses confirmed strong separation of community structures between UVJ and vagina, with more pronounced differences under unweighted Bray–Curtis distances, indicating that rare taxa contributed disproportionately to anatomical divergence. This aligns with prior reports in chickens and turkeys showing site-specific microbial communities along the reproductive tract (Lee et al., 2019; Shterzer et al., 2020; Kursa et al., 2022).

Previous studies in layer chickens reported *Firmicutes*, *Proteobacteria*, and *Cyanobacteria* as dominant phyla in the avian reproductive tract (Su et al., 2021b). Similarly, in broiler breeders of the Cobb breed at 37 weeks of age (Shterzer et al., 2020) and in 32-week-old laying SPF hens (Lee et al., 2019), *Firmicutes* was identified as the predominant phylum. Slightly different results were observed in the turkey oviduct, where the dominant phyla were *Proteobacteria*, *Firmicutes*, *Actinobacteria*, and *Bacteroidetes* (Kursa et al., 2022). Comparing the bacterial composition of turkey oviducts with that of chickens, certain phyla in low abundance, such as *Planctomycetes*, were detected in both species (Lee et al., 2019). In addition, *Firmicutes* and *Proteobacteria* were identified as the dominant phyla in the turkey respiratory tract (Kursa et al., 2021). Other low-abundance phyla, including *Fusobacteria*, *Cyanobacteria*, *Acidobacteria*, *Chloroflexi*, and *Deinococcus-Thermus*, have also been reported in the avian respiratory tract (Shabbir et al., 2015). Notably, these phyla *Firmicutes*, *Proteobacteria*, *Thermoproteota*, and *Actinobacteriota* were the dominant microbiota in turkey lower reproductive tract in this study.

Taxonomic profiling highlighted tissue-specific colonization patterns. Aamples from the UVJ were enriched in *Staphylococcus* and *Escherichia* ASVs, whereas VAG samples contained higher abundances of *Romboutsia_B.ilealis*, *Ligilactobacillus*, and *Limosilactobacillus*. The predominance of lactobacilli in the vagina is consistent with their well-documented role in producing lactic acid and antimicrobial compounds that maintain a protective, low-pH environment, thereby restricting colonization by opportunistic pathogens (Boskey et al., 2001; Ravel et al., 2011; Su et al., 2021a). These taxonomic differences are in line with previous reports describing site-specific microbial communities along the avian reproductive tract, with the UVJ acting as a selective niche for sperm storage and protection (Holt and Fazeli, 2016; Shterzer et al., 2024). Comparable patterns of anatomical specialization have also been observed in broiler breeders, where distinct bacterial assemblages were detected between the magnum and infundibulum, highlighting the influence of localized microenvironments on shaping microbial composition (Wen et al., 2021; Shterzer et al., 2024). Beyond the dominant taxa in the turkey lower reproductive tract, several low-abundance genera and species exhibited tissue-specific enrichment, suggesting potential niche-specific functions. In the VAG, these included *Anaerococcus*, *Clostridium*, *Ruminococcaceae*, *Preptostreptococcaceae_256921* (*Firmicutes_A*), *Limosilactobacillus. ingluviei*, *Enterococcus* (*Firmicutes_D*), and *UBA6249* (*Omnitrophota*). In the UVJ, enrichment was observed for *Limosilactobacillus fermentum* (*Firmicutes_D*), *UTCFX2. sp002050125* (*Chloroflexota*), and *UBA6902* (*Nitrospirota*). Although many of these taxa have not been previously reported in avian reproductive tissues, studies in other species indicate potential roles in mucosal metabolism, colonization resistance, and biofilm formation. For example, *Anaerococcus* (*Firmicutes_A*) has been reported in the vaginal microbiota of sows, where its abundance was associated with reproductive tract conditions such as pelvic organ prolapse during late gestation (Kiefer et al., 2021). While the host species differ, the repeated detection of *Anaerococcus* in mammalian and avian reproductive tracts suggests a conserved potential function in maintaining mucosal homeostasis or modulating local microbial communities. *Clostridium* and *Ruminococcaceae* (*Firmicutes_A*) are known SCFA producers in humans and bovines, which can lower vaginal pH and inhibit pathogen growth (Fusco et al., 2023; Gupta et al., 2024). *Limosilactobacillus. fermentum* (*Firmicutes_D*) contributes to mucosal protection and pathogen inhibition in mice and humans (Rodríguez-Sojo et al., 2021; Kang et al., 2018), while *Enterococcus* (*Firmicutes_D*) have been associated with nutrient metabolism and environmental adaptation in diverse hosts (Gupta et al., 2024).

Bardoxolone methyl administration did not significantly alter overall alpha diversity in either UVJ or vagina, consistent with a lack of broad community disruption. However, in the UVJ, unweighted beta diversity analyses revealed modest but significant separation between BAR and control groups, suggesting selective shifts in low-abundance taxa. Indeed, indicator species analysis identified increased *Corynebacterium* in bardoxolone methyl-treated birds, while *Pseudomonadaceae* and *Burkholderia* were more abundant in the controls. These findings are noteworthy because certain members of *Pseudomonas* and *Burkholderia* are known opportunists in avian reproductive systems and egg contents (Bassols et al., 2016; Eraky et al., 2020; Abd El-Ghany, 2021; Echols and Speer, 2022; Shterzer et al., 2024). Thus, bardoxolone methyl may promote a microbial environment less dominated by opportunistic taxa while favoring mucosa-adapted commensals such as *Corynebacterium*. In the vagina, neither alpha nor beta diversity metrics differed between groups, but BAR selectively enriched two rare taxa, *DTIZ01. sp011370625* (*Armatimonadota*) and *UBA6249* (*Omnitrophota*), paralleling the UVJ pattern of subtle selection on low-abundance members.

Predicted microbial pathways and genes revealed both shared core pathways and site-specific genes along the turkey lower reproductive tract. Core pathways, including the pentose phosphate pathway, adenosine *de novo* biosynthesis, and the Calvin-Benson-Bassham cycle, were abundant across both the UVJ and vagina, reflecting fundamental microbial energy metabolism and nucleotide biosynthesis (Stincone et al., 2015). Indicator pathways enriched in the vagina, such as dAMP synthesis II, Coenzyme A biosynthesis I, and glycolysis, suggest enhanced microbial proliferation, energy metabolism, and adaptation to anaerobic conditions, while UVJ-enriched genes, including sarcosine oxidase and choline dehydrogenase, support one-carbon metabolism, osmoprotection, and oxidative stress adaptation (Wu, 2009), potentially facilitating sperm storage. Bardoxolone methyl did not broadly alter overall pathway or gene diversity but selectively enriched functions related to energy metabolism, nucleotide biosynthesis, and regulatory systems for environmental sensing, consistent with microbiome functional resilience, in which microbes maintain core functions under fluctuating conditions without major shifts in community composition (Lozupone et al., 2012). In the UVJ, BAR enhanced carbohydrate and nucleotide metabolism along with regulatory pathways, including NAD and isoprenoid biosynthesis, peptidoglycan biosynthesis, the ethylmalonyl-CoA pathway, consisting of Cell cycle transcriptional regulator A (CtrA), Cell cycle histidine kinase A (CckA), Histidine phosphotransferase ChpT, Cell cycle phosphodiesterase regulator CpdR, Chromosomal virulence sensor kinase ChvG, and Chromosomal virulence response regulator ChvI, indicating improved microbial adaptability to host signals, cell cycle control, and stress resilience (Hooper et al., 2012; Belkaid and Hand, 2014; Bhagat et al., 2025). In the vagina, BAR selectively enriched metabolic pathways such as menaquinone biosynthesis and polyisoprenoid biosynthesis, as well as genes including *pyruvate dehydrogenase*, *hydrogenase A*, and *betaine aldehyde dehydrogenase*, supporting energy production, redox balance, nucleotide turnover, protein homeostasis, and carbon substrate flexibility, thereby promoting microbial metabolic robustness, stress adaptation, and community maintenance (Meganathan and Kwon, 2009). Overall, these findings suggest that BAR reinforces microbiome functional resilience and mucosal stability without broadly altering composition, which may support genomic stability and protein homeostasis.

Our findings also highlight that avian reproductive tract microbiomes differ fundamentally from those in mammals. While human vaginal microbiota is typically dominated by *Lactobacillus* species (Ravel et al., 2011), our profiles confirm previous observations that the avian reproductive tract harbors a more diverse community of *Firmicutes, Proteobacteria, Actinobacteriota*, and other taxa (Lee et al., 2019; Glendinning et al., 2020). This emphasizes the need for species-specific perspectives when interpreting microbial contributions to the function of the reproductive tract.

## 5 Conclusion

In conclusion, administration of bardoxolone methyl subtly but consistently modulated the turkey lower reproductive tract microbiome. This modulation primarily involved enrichment of rare taxa and functional pathways related to metabolism, redox balance, and environmental sensing, without disrupting overall microbial diversity. These findings are consistent with known NRF2 biology and suggest that antioxidant-mediated redox regulation can fine-tune mucosal microbial communities, particularly in the UVJ. While the present manuscript focuses on the effects of BAR on the microbial communities within the UVJ and vagina, we have also evaluated its influence on gene and protein expression in these tissues, as well as its impact on key reproductive traits. The outcomes of these complementary analyses will be detailed in a separate manuscript. Future studies should integrate multi-omics approaches and reproductive performance metrics to validate the functional consequences of these microbial changes and to further elucidate host–microbe interactions relevant to fertility in poultry.

## Data Availability

All sequencing data supporting the findings of this study have been deposited in the NCBI Sequence Read Archive (SRA) under the BioProject accession number PRJNA1311734 and are available at http://www.ncbi.nlm.nih.gov/bioproject/1311734.
