# Supplementary figures and images for: Effect of bardoxolone methyl on the lower reproductive tract microbiome in turkey breeder hens
Source: Front Physiol. 2025 Nov 17;16:1703742. doi: 10.3389/fphys.2025.1703742 (PMC12665593; doi:10.3389/fphys.2025.1703742)

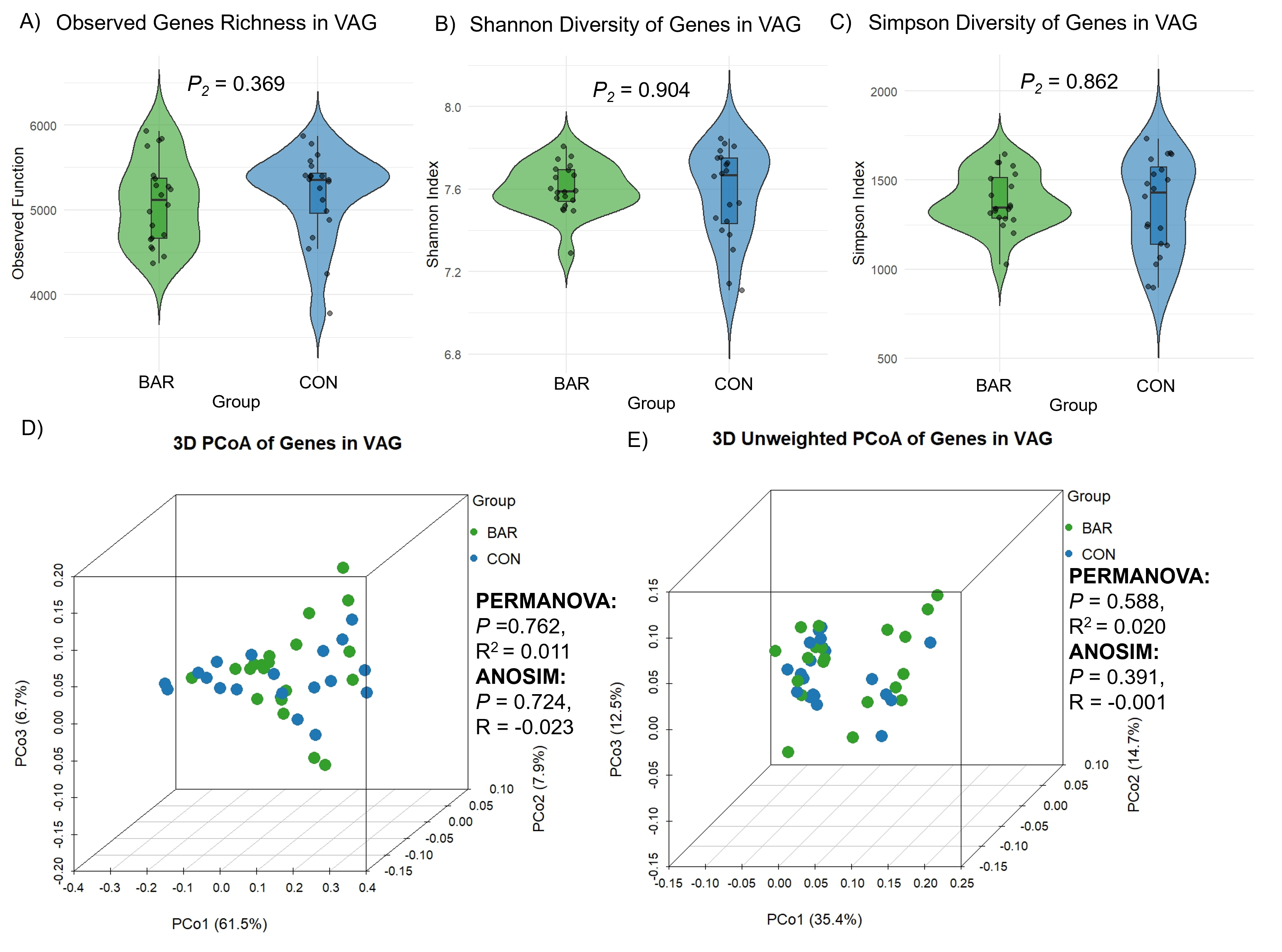

Supplement: Supplementary file 1 [file Image6.tif]

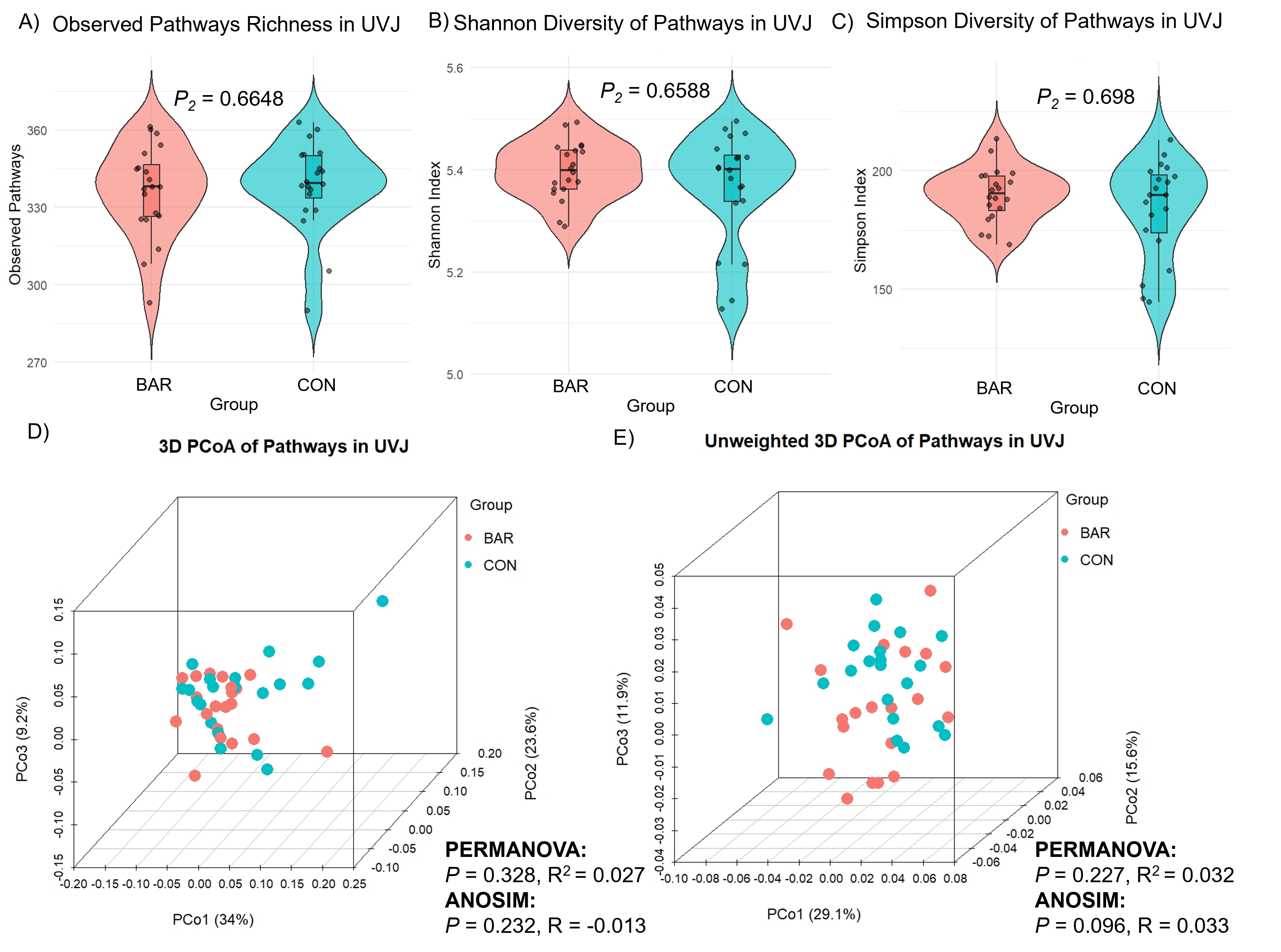

Supplement: Supplementary file 2 [file Image3.tif]

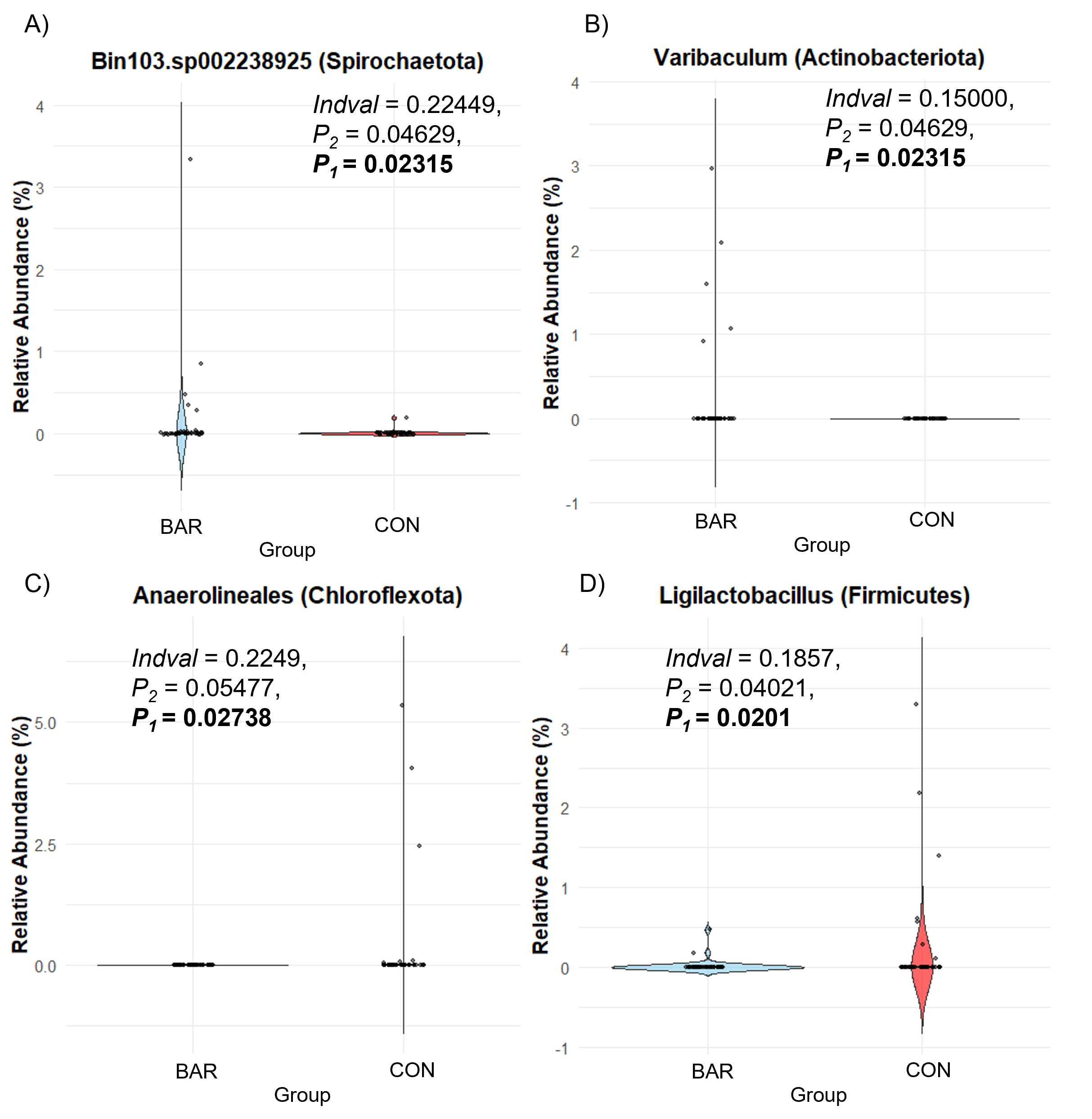

Supplement: Supplementary file 3 [file Image2.tif]

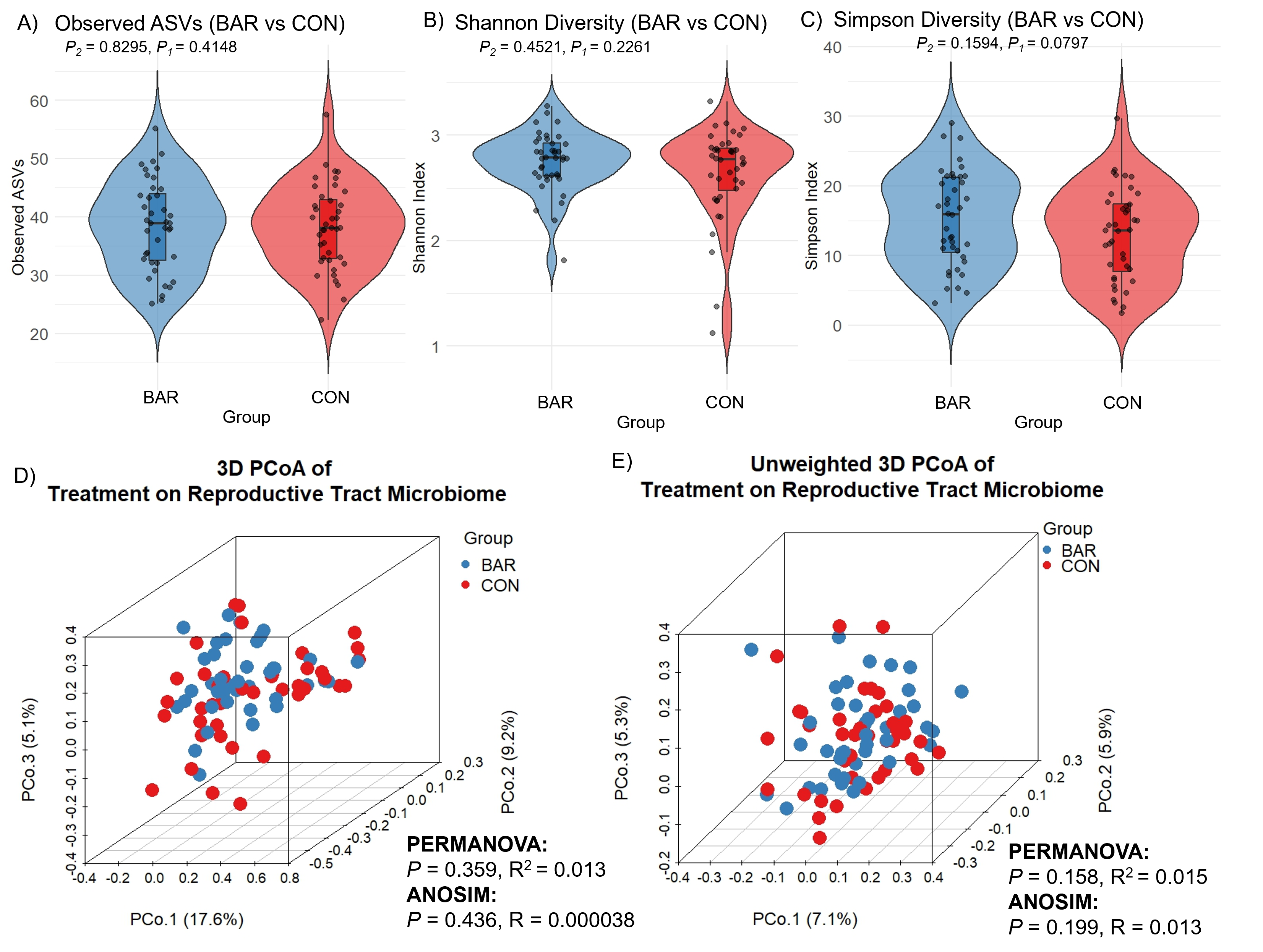

Supplement: Supplementary file 4 [file Image1.tif]

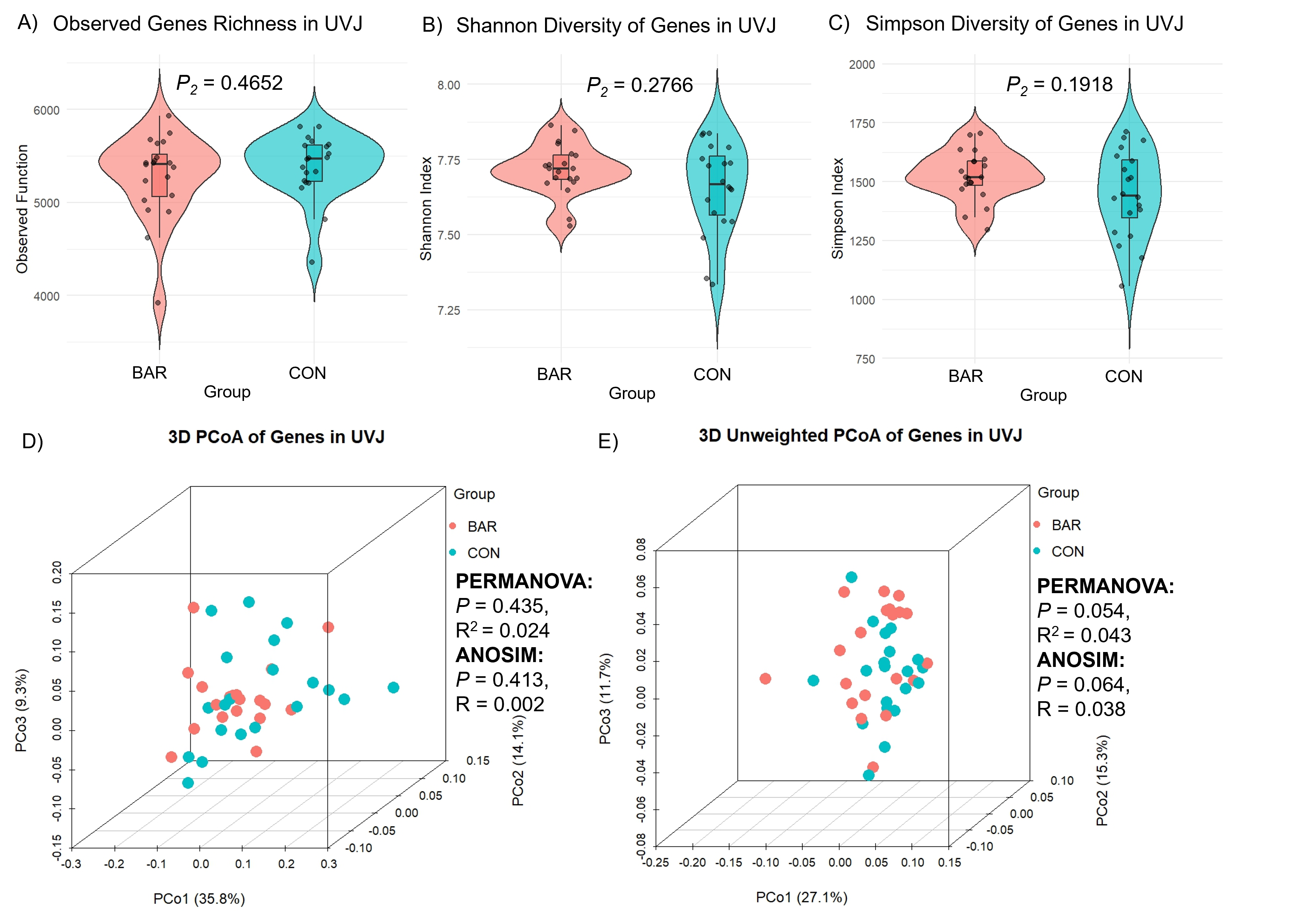

Supplement: Supplementary file 5 [file Image4.png]

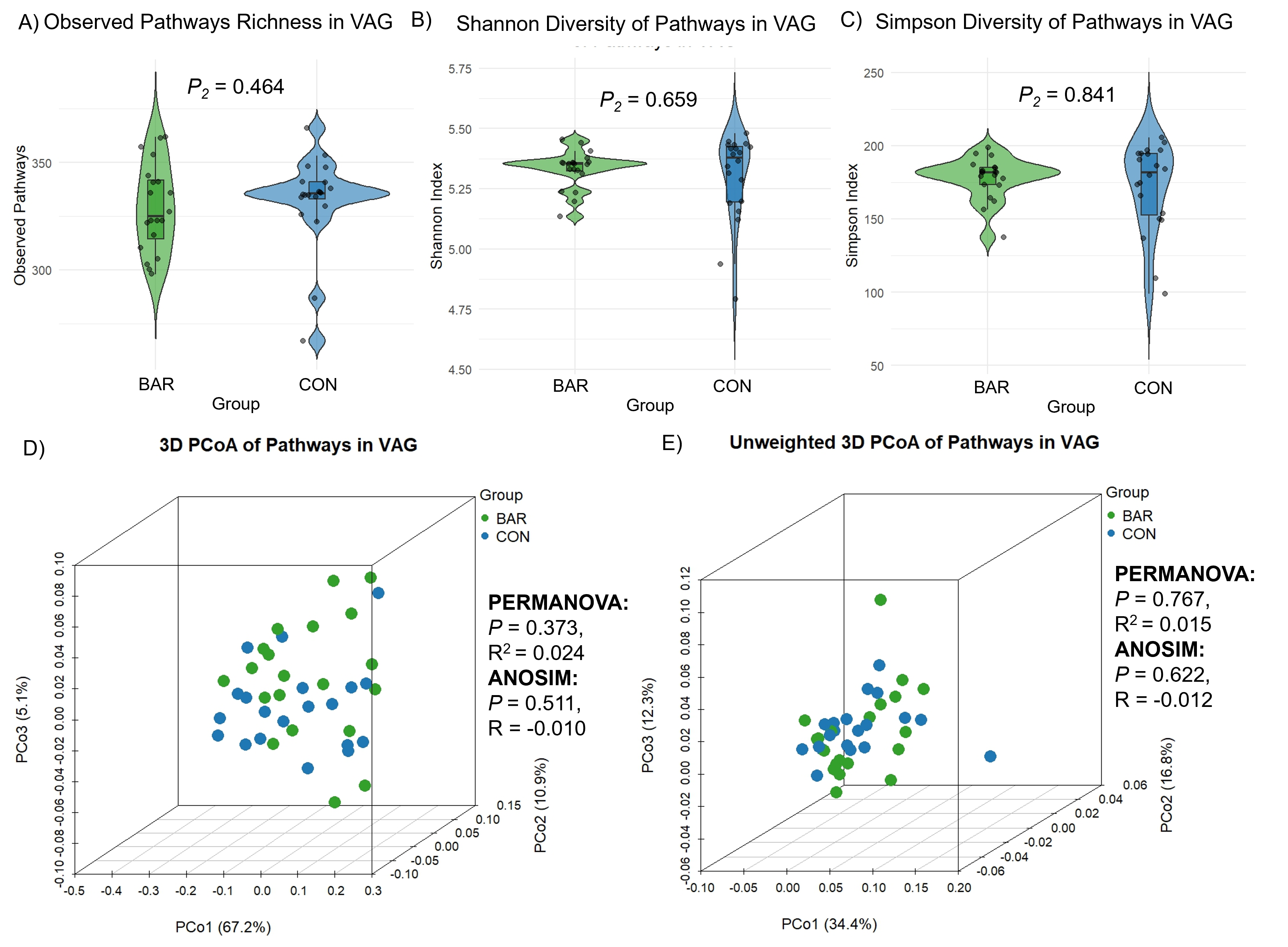

Supplement: Supplementary file 6 [file Image5.tif]
